# Wetland Restoration Effects on Waterbird Diversity and Habitat Use: A Long-Term Case Study from Chongming Dongtan in Shanghai, China

**DOI:** 10.3390/biology15120926

**Published:** 2026-06-13

**Authors:** Baodong Yuan, Dongmei Li, Yeai Zou, Xiaoteng Shen

**Affiliations:** 1 College of Agriculture and Biology, Liaocheng University, Hunan Road, Liaocheng 252000, China; yuanbao365@163.com; 2 National Field Scientific Observation and Research Station of Dongting Lake Wetland Ecosystem in Hunan Province, Institute of Subtropical Agriculture, Chinese Academy of Sciences, Changsha 410125, China; 3 Technology Innovation Center for Ecological Conservation and Restoration in Dongting Lake Basin, Ministry of Natural Resources, Changsha 410007, China; 4 University of Chinese Academy of Sciences, Beijing 100049, China; 5 State Key Laboratory of Water Disaster Prevention, Hohai University, Nanjing 210024, China

**Keywords:** wetland restoration, waterbirds, habitat use, seasonal variation, spillover effect, Chongming Dongtan

## Abstract

The continued loss and degradation of wetlands pose major challenges to global waterbird conservation. In response, large-scale wetland restoration projects have been widely implemented worldwide, yet their long-term ecological effectiveness has not been sufficiently evaluated. Here, we assessed the long-term impacts of wetland restoration on waterbird communities at Chongming Dongtan Wetland, China, using 17 years of monitoring data spanning pre-restoration, restoration, and post-restoration phases. Our results suggest that the Ecological Control of *Spartina alterniflora* and Improvement of Bird Habitats substantially enhanced waterbird diversity, with both species richness and total abundance increasing significantly after restoration. Restored artificial wetlands supported particularly high abundances of waterbirds, confirming their role as critical supplementary habitats alongside natural tidal flats. Notably, different waterbird guilds exhibited pronounced seasonal shifts in habitat use: the Anatidae predominated during the wintering period, whereas Waders dominated during spring and autumn migrations, and the degree of reliance on artificial versus natural wetlands varied markedly between guilds and across seasonal cycles. Beyond local effects, we detected a clear spillover effect, whereby increases in waterbird abundance and species richness were also observed in adjacent non-restored natural intertidal mudflats following restoration. In addition, several threatened and nationally protected species were recorded exclusively during the post-restoration phase, indicating improved habitat suitability for conservation-priority taxa. Overall, our findings highlight that wetland restoration can generate both local and landscape-scale biodiversity benefits, emphasizing the importance of incorporating habitat heterogeneity, seasonal habitat requirements, and spillover effects into coastal wetland restoration and management strategies.

## Simple Summary

The continued loss and degradation of wetlands pose major challenges to global waterbird conservation. We assessed the long-term impacts of wetland restoration on waterbird communities at Chongming Dongtan Wetland, China, using 17 years of monitoring data spanning pre-restoration, restoration, and post-restoration phases. Our results suggest that the Ecological Control of *Spartina alterniflora* and Improvement of Bird Habitats substantially enhanced waterbird diversity, with both species richness and total abundance increasing significantly after restoration.

## 1. Introduction

Wetland degradation and loss pose profound threats to waterbird diversity. With billions of migratory birds traveling thousands of kilometers annually between breeding and non-breeding grounds [1], both long- and short-distance migrants depend on intact wetland networks to complete their life cycles [2]. Since the 1950s, China has experienced extensive and sustained wetland loss [3,4,5], resulting in a steep and continuous decline in waterbird diversity [6,7]. Over 100 species of birds in Asia are threatened by wetland degradation, loss, and fragmentation [8,9,10], with 84 out of 260 waterbird species in China showing a downward population trend [11].

Wetland restoration is a crucial strategy for mitigating the current biodiversity crisis. China has implemented numerous ecological conservation and restoration projects [12,13], which have consistently enhanced biodiversity and ecosystem services, promoting sustainable development [14]. Successful ecological restoration projects not only enhance biodiversity within target patches but also produce spillover effects at the landscape scale, boosting biodiversity in surrounding non-target areas. Studies have shown that such effects extend beyond biodiversity, impacting ecosystem goods [15,16] and services [17]. For instance, restoration projects for urban rivers and mining subsidence lakes have generated significant positive spillover effects on surrounding property values [15]. Given the widespread occurrence of these spillover events, assessing the success of a restoration project requires expanding the assessment scope beyond the physical boundaries of the project site to encompass landscape-scale outcomes [16].

Waterbirds are sensitive bioindicators of wetland ecosystem health, as their population dynamics reflect habitat quality and food availability [7,18]. This high sensitivity to habitat conditions renders waterbirds a highly valuable bioindicator group in ecological research and conservation practice. Numerous studies have demonstrated that when wetlands are disturbed or degraded, changes in waterbird diversity, abundance, and population structure occur rapidly, providing early warnings of ecosystem function decline or improvement [19]. Therefore, systematic monitoring of waterbird communities is crucial for evaluating the outcomes of wetland restoration, ecological succession, and management effectiveness [8,9,20,21,22]. This bioindicator value is reflected not only in the responses of waterbirds to changes in habitat quality, vegetation structure, and hydrological regimes, but also in their feedback effects to changes in wetland landscape dynamics, making waterbird monitoring an indispensable, core component of wetland ecological restoration projects [23,24].

China’s coastal wetlands are critical stopover and wintering grounds along the East Asian–Australasian flyway [25]. Several threatened migratory waterbirds, including shorebird species along the East Asian–Australasian Flyway, rely heavily on coastal wetlands in eastern China [26]. Chongming Dongtan Wetland is one of China’s most important coastal wetland ecosystems. However, the invasive *Spartina alterniflora* has caused severe ecological degradation of the wetland [24]. This species was initially introduced to China in 1993 for coastal protection, erosion control, and siltation mitigation [26]. However, its rapid range of expansion has suppressed the growth of other species, and *Spartina alterniflora* has gradually replaced native plant communities (e.g., *Scirpus mariqueter* and reed), becoming one of the dominant species in the intertidal zone [25]. The invasion of *Spartina alterniflora* has led to a reduction in the diversity and abundance of bird food resources, such as large benthic invertebrates [27], which has, in turn, altered waterbird community composition and resulted in a significant reduction in bird populations within the reserve [28]. To restore habitats, the reserve implemented various conservation and restoration efforts, with the Ecological Control of *Spartina alterniflora* and Improvement of Bird Habitats being the most extensive in terms of restoration area and implementation effort [26,27]. Previous studies have shown that coastal wetland restoration can improve waterbird diversity in the short term [28], but the long-term ecological benefits of such projects have not been sufficiently evaluated [29,30].

Despite a growing body of evidence demonstrating that coastal wetland restoration delivers measurable benefits to waterbird communities, most existing studies have focused on short-term responses or single-habitat types, leaving the long-term, landscape-scale ecological effects of restoration poorly understood. In particular, it remains unclear how different waterbird guilds respond to restoration across seasons, how restored artificial wetlands interact with surrounding natural tidal flats, and whether site-specific restoration actions can generate biodiversity spillover effects beyond physical boundaries of restoration projects.

Seventeen years of continuous monitoring data from the Chongming Dongtan Wetland were used; this study evaluates the long-term ecological effects of wetland restoration by comparing waterbird community dynamics before, during, and after the implementation of large-scale *Spartina alterniflora* control and habitat improvement. Specifically, we examine (1) whether wetland restoration increases waterbird species richness and abundance over the long term; (2) whether different waterbird guilds exhibit distinct seasonal patterns of habitat use between artificial wetlands and natural mudflats; and (3) whether restoration benefits extend to adjacent, non-restored habitats through landscape-scale spillover effects. By addressing these questions, our study provides empirical evidence to inform coastal wetland restoration and waterbird conservation strategies under intensifying anthropogenic pressure.

## 2. Methods

### 2.1. Study Area

**Figure 1:**
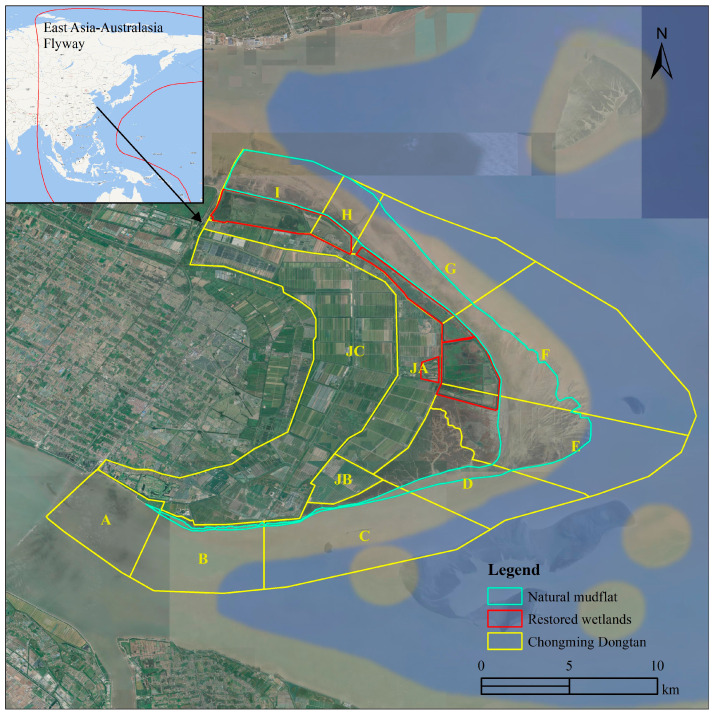
Location of Chongming Dongtan National Nature Reserve. The restored wetlands refer to the Ecological Control of *Spartina alterniflora* and Improvement of Bird Habitats project.

Chongming Dongtan National Nature Reserve is located at the easternmost tip of Chongming Island at the mouth of the Yangtze River (121°43′–122°05′ E, 31°24′–31°39′ N). It serves as a key wintering and stopover site for migratory waterbirds along the East Asia–Australasia flyway, playing a crucial role in maintaining the diversity of waterbirds in the Yangtze River Basin and globally [20]. However, the invasion of *Spartina alterniflora* has posed severe threats to bird habitats and food resources. To restore degraded habitats, the Shanghai Municipal Government initiated the Ecological Control of *Spartina alterniflora* and Improvement of Bird Habitats in September 2013, which was nearing completion by 2017. This restoration project aimed to enhance habitat diversity to meet the needs of various bird species, including both natural tidal wetlands and artificial wetlands, such as fish ponds and restored areas. The project area extends annually in the direction of the sea due to sediment deposition from the Yangtze River. The study area encompasses 80% of the core natural wetlands and all the artificial wetlands within Chongming Dongtan National Nature Reserve (Figure 1). Specifically, the core natural wetland refers to the key intertidal mudflat area of the nature reserve, while the artificial wetlands are mainly composed of ecological restoration areas and wetland parks.

### 2.2. Waterbird Data

Field surveys were conducted annually from 2007 to 2023, covering the pre-restoration (2007–2013), restoration (2014–2016), and post-restoration (2017–2023) phases. Annual waterbird surveys were conducted from November to October of the following year, with 16–18 surveys per year, depending on weather conditions. Surveys were conducted around the mid-tide mark each month, with two surveys in the peak migration seasons (spring and autumn) and one survey per month in other periods. Surveys used binoculars (10×) and monoculars (20–60×) to record all waterbird species and counts in designated study areas covering 80% of the core natural wetlands and more than 90% of the artificial wetlands (Figure 1).

The bird survey area covers approximately 80% of the core mudflat area of the reserve (D, E, F, G) and more than 90% of the artificial wetlands in the Dongtan International Important Wetland. The artificial wetlands in the Dongtan area, which are particularly important for waterbird habitats, mainly consist of the Shanghai Dongtan Wetland Park, the newly established Ecological Control of *Spartina alterniflora* and Improvement of Bird Habitats, as well as shrimp and crab farming ponds (Figure 1). It is important to note that due to changes in land use types and the implementation of restoration projects, the area of artificial wetlands in this study varied before and after the restoration. In the pre-restoration phase, the artificial wetlands only included fish and crab farming ponds, whereas after the restoration project was implemented, the area of artificial wetlands increased (Figure 1), though the total study area remained consistent. The study timeline is divided into three phases based on the implementation of the *Spartina alterniflora* Ecological Control and bird habitat optimization project: pre-restoration (*n* = 7 years), restoration phase (*n* = 3 years), and post-restoration phase (*n* = 7 years).

### 2.3. Statistical Analysis of Data

Due to significant seasonal differences in bird communities, we analyzed bird data separately for each season. According to the migration and wintering patterns of waterbirds in Chongming Dongtan wetland, seasons were divided into spring (northward migration period, from March to May), autumn (southward migration period, from August to October) and winter periods (overwintering period, from November to February). Multiple surveys were conducted each year in the same area, with the number of surveys and survey times generally kept consistent. Therefore, the number of bird survey events was recorded and we calculated the average number of species, average individual counts, and relative abundance for each season during the pre-restoration, restoration, and post-restoration periods in the study area for further analysis. We also separately calculated the changes in the proportion of bird species in artificial and natural wetlands across different restoration phases.

Waterbirds were classified into four groups based on their habitat preferences and ecological habits: (1) Anatidae, which are typically swimming birds that forage in water (e.g., geese, ducks, and swans); (2) Waders, which are small wading birds that forage in shallow water and moist soil (e.g., shorebirds); (3) Gulls; and (4) Herons, which are larger wading birds that typically forage in shallow water and moist soil (e.g., Herons and egrets). We identified the species and relative abundance of each group of waterbirds across different seasons and restoration phases in both natural and artificial wetlands, assessing the impact of restoration on the species richness and abundance of specific bird groups. We defined the spillover effect as a significant increase in waterbird abundance in non-restored natural mudflats during the post-restoration phase, after accounting for survey effort, compared with the pre-restoration baseline [27].

Bird species richness was log-transformed using the formula log(x + 1) prior to analysis. Density was quantified as the number of individuals per hectare to represent species abundance. Waterbird density in artificially restored wetlands was calculated using the total post-restoration area, including previously degraded areas invaded by *Spartina alterniflora*. This standardization controlled for the effect of area expansion, allowing us to isolate the impact of improved habitat quality on waterbird abundance. Differences in birds among restoration phases (pre-restoration, recovery, and post-restoration) were examined using a one-way Kruskal–Wallis rank-sum test due to non-normal data distribution. Omega-squared correction was applied when calculating eta-squared effect sizes to quantify the magnitude of phase-related differences. When the Kruskal–Wallis test indicated significant effects (*p* < 0.05), post hoc pairwise comparisons were conducted using Dunn’s test with Holm correction for multiple testing.

Detection frequency was used to assess the effectiveness of the ecological restoration project in protecting nationally protected and threatened bird species (those listed as critically endangered (CR), endangered (EN), vulnerable (VU), or near-threatened (NT) in the IUCN Red List) by monitoring their occurrence before, during, and after wetland restoration. Detection frequency was defined in two complementary ways, i.e., (1) species-level detection frequency, which is the number of years a given threatened species was recorded during each restoration phase, and (2) annual detection frequency, which is the total number of threatened species recorded in a single survey year. For statistical comparison, a species was classified as “detected” in a phase if it was recorded in at least one year of that phase. Fisher’s exact test (two-tailed) was used to compare detection rates of threatened species between each pair of restoration phases (pre-restoration vs. restoration, restoration vs. post-restoration, and pre-restoration vs. post-restoration).

All statistical analyses were performed in R (version 4.4.2; R Core Team, 2025) [31]. The R packages ggplot2 [32], dplyr [33], FSA [34], and rcompanion [35] were used for data manipulation, visualization and statistical tests.

## 3. Results

### 3.1. Patterns of Bird Population in Different Recovery Periods

**Figure 2:**
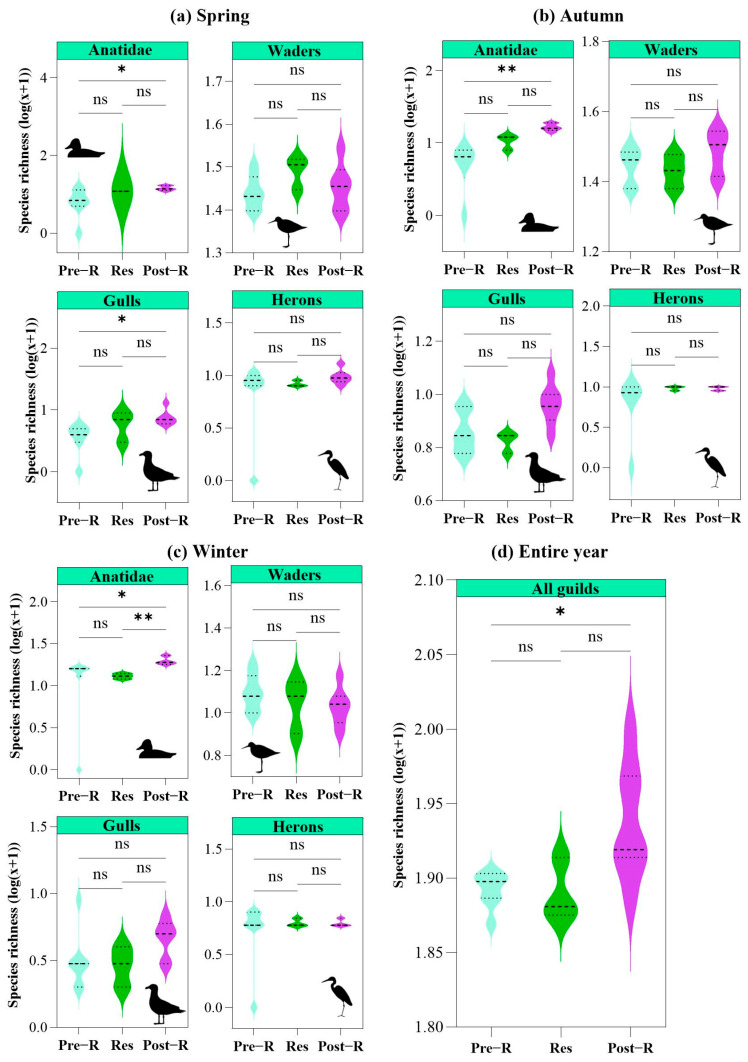
Variation in individual abundance of different bird groups across restoration periods (Pre−R: 2007–2013; Res: 2014–2016; Post−R: 2017–2023. * *p* < 0.05, ** *p* < 0.01, ns *p* > 0.05).

The species richness and population from 2007 to 2023 in Chongming Dongtan National Nature Reserve are shown in Figure S1. During the northward migration period in spring, the densities of both Anatidae (H = 10.22, df = 2, *p* < 0.01) and Herons (H = 10.85, df = 2, *p* < 0.01) differed significantly among restoration periods. Specifically, their densities were significantly higher in later restoration stages compared to the initial stage (Anatidae: Z = 3.19, *p* < 0.01; Herons: Z = 3.21, *p* < 0.01; Figure 2a). During the southward migration period in autumn, the densities of Anatidae (H = 11.21, df = 2, *p* < 0.01), Waders (H = 11.67, df = 2, *p* < 0.01), Gulls (H = 11.67, df = 2, *p* < 0.01), and Herons (H = 9.12, df = 2, *p* < 0.05) all differed significantly across restoration periods. Subsequent comparisons indicated that densities of all four groups were significantly higher in post-restoration stages compared to pre-restoration (Anatidae: Z = 3.15, *p* < 0.01; Waders: Z = 3.42, *p* < 0.01; Gulls: Z = 3.42, *p* < 0.01; Herons: Z = 2.79, *p* < 0.05; Figure 2b). During the wintering period, only the density of Anatidae (Z = 3.33, *p* < 0.01, Figure 2c) was significantly higher than that in the pre-restoration phase. At the community level, both the density and the species richness of all waterbirds differed significantly among restoration stages (abundance: H = 12.72, df = 2, *p* < 0.01; richness: H = 7.88, df = 2, *p* < 0.05), with significantly higher values recorded in the post-restoration period compared with the pre-restoration baseline (Density: Z = 3.55, *p* < 0.01; Figure 2d and Figure S2d).

**Figure 3:**
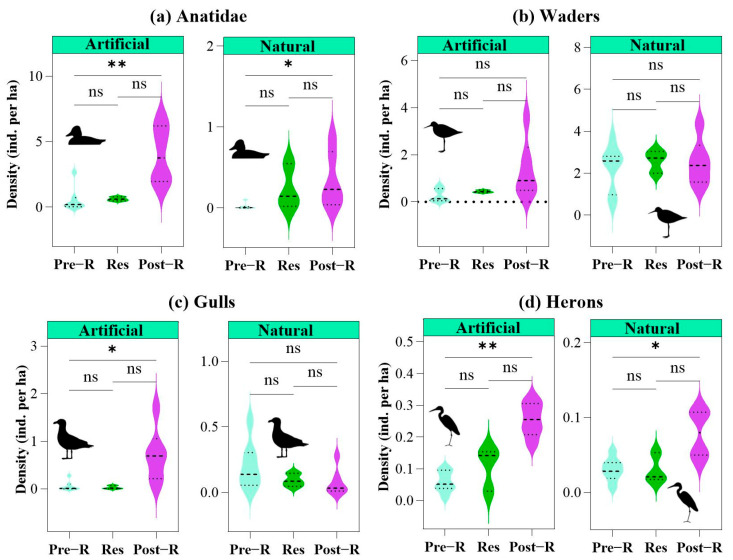
Variation in individual abundance of different bird groups across restoration periods in spring (Pre−R: 2007–2013; Res: 2014–2016; Post−R: 2017–2023. * *p* < 0.05, ** *p* < 0.01, ns *p* > 0.05).

During the spring northward migration period, in artificial wetlands, results revealed significant differences in the densities across restoration periods for Anatidae (H = 9.73, df = 2, *p* < 0.01), Waders (H = 6.25, df = 2, *p* < 0.05), Gulls (H = 9.25, df = 2, *p*< 0.01), and Herons (H = 11.014, df = 2, *p* < 0.01). Subsequent tests showed that their densities were significantly higher in the post-restoration stage compared to the pre-restoration stage (Anatidae: Z = 3.08, *p* < 0.01, Figure 3a; Waders: Z = 2.39, *p* < 0.05, Figure 3b; Gulls: Z = 2.76, *p* < 0.05, Figure 3c; Herons: Z = 3.26, *p* < 0.05, Figure 3d). In natural wetlands, however, significant differences were only detected for the sensitivities of Anatidae (H = 9.29, df = 2, *p* < 0.01) and Herons (H = 7.37, df = 2, *p* < 0.05). Notably, the effect sizes (*η*^2^) were lower than those in artificial wetlands (Anatidae: artificial wetlands: natural wetlands = 0.59:0.56; Herons: artificial wetlands: natural wetlands = 0.69:0.41; Table 1). Similarly, the densities of these two groups were significantly higher in the post-restoration than pre-restoration stages (Anatidae: Z = 2.92, *p* < 0.01, Figure 3a; Herons: Z = 2.46, *p* < 0.05, Figure 3d).

**Figure 4:**
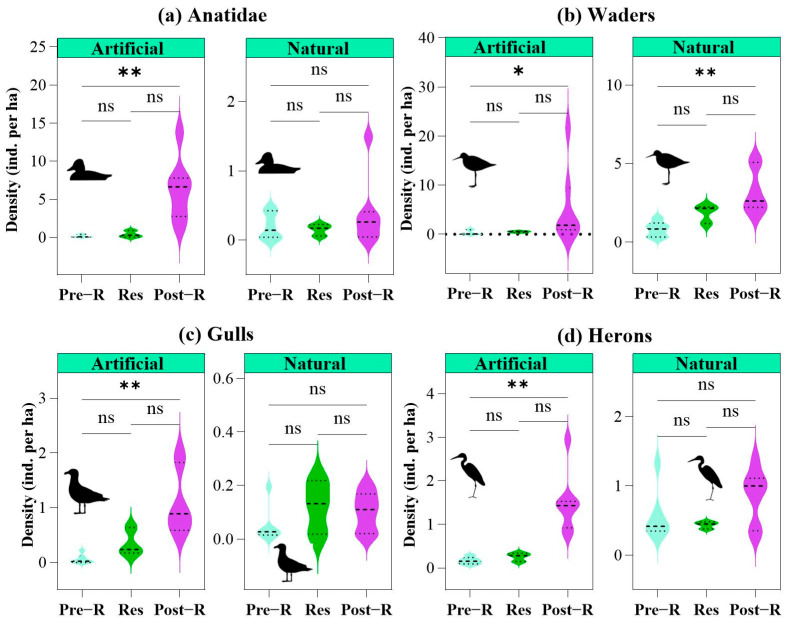
Variation in individual abundance of different bird groups across restoration periods in autumn (Pre−R: 2007–2013; Res: 2014–2016; Post−R: 2017–2023. * *p* < 0.05, ** *p* < 0.01, ns *p* > 0.05).

During the autumn southward migration period, in artificial wetlands, the results of tests indicated significant differences in densities across restoration periods for Anatidae (H = 11.32, df = 2, *p* < 0.01), Waders (H = 8.63, df = 2, *p* < 0.05), Gulls (H = 11.69, df = 2, *p* < 0.01), and Herons (H = 11.67, df = 2, *p* < 0.01). As shown in the left panels of Figure 4a–d, test results revealed that the densities were significantly higher in the post-restoration stage compared to the pre-restoration stage (Anatidae: Z = 3.21, *p* < 0.01, Figure 4a; Waders: Z = 2.81, *p* < 0.05, Figure 4b; Gulls: Z = 3.42 *p* < 0.01, Figure 4c; Herons: Z = 3.34, *p* < 0.01, Figure 4d). In contrast, within natural wetlands, a significant difference was found only for the density of Waders (H = 11.31, df = 2, *p* < 0.01). Accordingly, their densities in the post-restoration period were significantly higher than the pre-restoration level (Z = 3.36, *p* < 0.01, Figure 4b).

**Figure 5:**
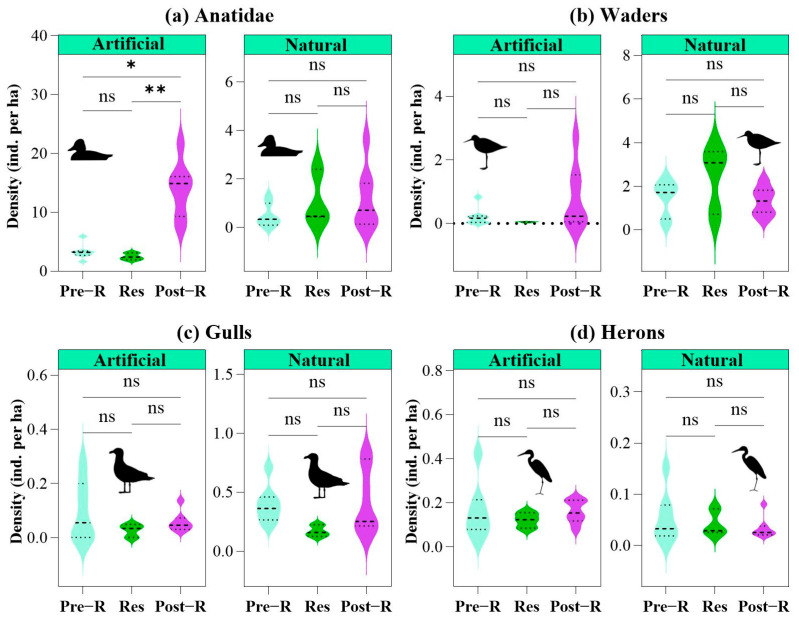
Variation in individual abundance of different bird groups across restoration periods in winter (Pre−R: 2007–2013; Res: 2014–2016; Post−R: 2017–2023. * *p* < 0.05, ** *p* < 0.01, ns *p* > 0.05).

During the overwintering period, a significant difference in the densities across restoration periods was found only for Anatidae in artificial wetlands (H = 12.23, df = 2, *p* < 0.01). Specifically, their density was higher for the post-restoration rather than the pre-restoration level (Z = 2.86, *p* < 0.01, Figure 5a).

### 3.2. Patterns of Bird Distribution in Different Restoration Periods

**Figure 6:**
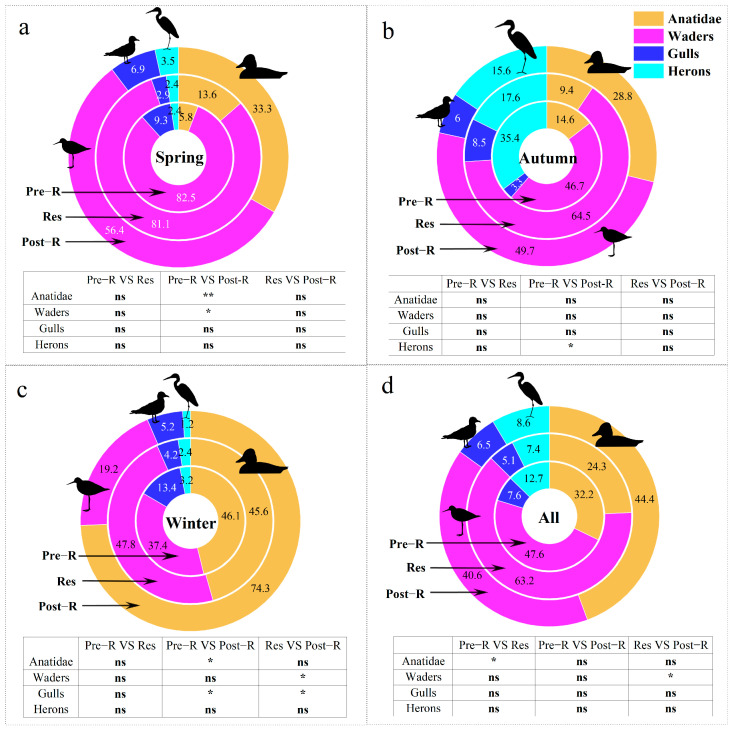
Changes in the proportion of different bird groups across restoration periods (Pre−R: 2007–2013; Res: 2014–2016; Post−R: 2017–2023. * *p* < 0.05, ** *p* < 0.01, ns *p* > 0.05). (**a**) The spring waterbird community structure; (**b**) The autumn waterbird community structure, (**c**) The winter waterbird community structure; (**d**) The total waterbird community structure.

The total waterbird community structure differed significantly among restoration phases (Figure 6d). The proportion of different waterbird guilds varies across seasons and restoration periods. During spring northward migration and autumn southward migration, shorebirds dominated the study area, with their proportion reaching 56.4–82.5% (Figure 6a) and 46.7–64.5% (Figure 6b), respectively. During the wintering period, Anatidae were dominant in the post-restoration phase but not in the restoration phase, and their proportion differed significantly among phases, ranging from 46.1% to 74.3% (Figure 6c). In the post-restoration phase, the proportion of Anatidae was significantly higher than in the pre-restoration phase during both the spring northward migration (Z = 3.29, *p* < 0.01, Figure 6a) and wintering period (Z = 2.58, *p* < 0.05, Figure 6c). However, during the spring northward migration, the proportion of shorebirds was significantly lower than in the pre-restoration phase (Z = −2.45, *p* < 0.05, Figure 6a). Habitat degradation, insufficient food resources and intensified interspecific competition suppressed the population development of Anatidae, leading to their low proportion in the waterbird community. The precise optimization of habitat structure, comprehensive recovery of food resources, elimination of anthropogenic disturbance, enhancement of habitat connectivity, and reduction in interspecific competition jointly drove a significant increase in the proportion of Anatidae populations, making them the absolutely dominant group in the study area.

**Figure 7:**
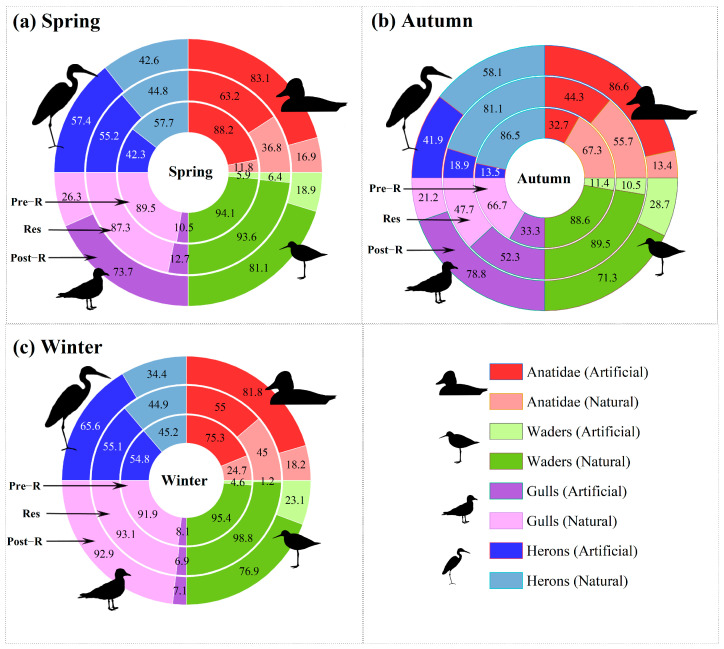
Changes in the proportion of bird groups in artificial wetlands and natural mudflats across different restoration periods (Pre−R: 2007–2013; Res: 2014–2016; Post−R: 2017–2023).

The relative abundance of different waterbird groups in artificial wetlands and natural mudflats varied across seasons and restoration periods. Most Anatidae occurred in artificial wetlands, whereas Waders were far more abundant on natural mudflats than in artificial wetlands (Figure 7). Herons showed a more even distribution between natural mudflats and artificial wetlands. Gulls were concentrated on natural mudflats during the wintering period (Figure 7c), but their use of artificial wetlands increased markedly during the autumn southward migration (Figure 7b). Compared with other seasons, Anatidae also increased their use of natural wetlands in autumn (Figure 7b). Herons relied more on artificial wetlands during the spring northward migration (Figure 7a) and wintering period (Figure 7c), but were more dependent on natural wetlands during the autumn southward migration (Figure 7b).

During the wintering period, the proportional composition of each group remained broadly similar between the pre- and post-restoration phases. In contrast, during the spring northward migration (Figure 7a) and autumn southward migration (Figure 7b), the proportional use of artificial wetlands increased in the post-restoration phase for Gulls, Herons, and shorebirds. Specifically, during spring migration, the proportion of Gulls in artificial wetlands increased from 10.5% in the pre-restoration phase to 73.7% after restoration (Figure 7a). During autumn migration, the proportion of Anatidae in artificial wetlands increased from 32.7% to 86.6%, and the proportion of Herons increased from 13.5% to 58.1% (Figure 7b).

### 3.3. Threatened Species Detection Frequency During Ecosystem Recovery

**Figure 8:**
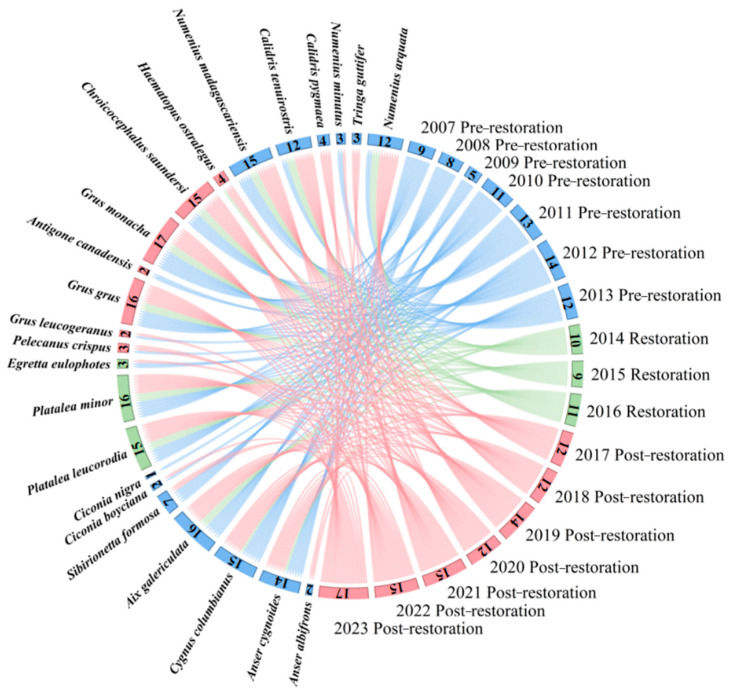
Detection frequency of rare and endangered species across restoration periods. Note: Detection frequency is shown in the right half of Figure 8. For example, the detection frequency was 9 in 2007, meaning 9 species of rare and endangered birds were detected that year. Detection frequency is shown in the left half of Figure 8. For instance, the detection frequency of Grus grus is 16, indicating this species was recorded over 16 years.

A total of 23 rare and threatened species were detected across the study period (2007–2023), with 19 species in the pre-restoration phase (2007–2013) and 21 species in the post-restoration phase (Figure 8). Fisher’s exact test showed no statistically significant difference in overall detection rates between pre- and post-restoration phases (two-tailed, *p* > 0.99). However, a net increase in threatened species richness was observed, i.e., four species were recorded exclusively in the post-restoration phase, while only two species were detected in the pre-restoration phase. Notably, the newly recorded species included three globally threatened taxa of high conservation priority, i.e., the Spoon-Billed Sandpiper (*Calidris pygmaea*, CR), Nordmann’s Greenshank (*Tringa guttifer*, EN), and Lesser White-Fronted Goose (*Anser albifrons*, VU). In contrast, the two species that were not detected post-restoration (Black Stork *Ciconia nigra* and Sandhill Crane *Antigone canadensis*) were both rare vagrants, each recorded only once during the entire monitoring period. This pattern suggests an overall improvement in habitat suitability for conservation-priority waterbirds following restoration.

To further evaluate the impacts of restoration activities on threatened species, we compared detection rates across the three phases using Fisher’s exact tests. During the restoration construction phase (2014–2016), the detection rate of threatened species decreased from 82.6% (pre-restoration) to 52.2%, with eight species temporarily absent from the survey records. Although this difference was not statistically significant (Fisher’s exact test, *p* = 0.3168), the relative risk of detection was 2.32 times higher in the pre-restoration phase than during restoration. Following the completion of restoration (post-restoration, 2017–2023), the detection rate rebounded strongly to 91.3%. Notably, all 12 species detected during the restoration phase were retained in the post-restoration phase, and 9 out of the 11 species absent during restoration returned to the study area. Our results suggest that the short-term disturbance caused by restoration construction to rare and threatened waterbirds is reversible, and the long-term ecological benefits far outweigh the transient negative impacts.

## 4. Discussion

### 4.1. Wetland Restoration Enhanced Waterbird Diversity

Consistent with previous studies [36,37,38], our results show that wetland restoration significantly increased both species richness and abundance, thereby supporting higher waterbird diversity (Figure 2, Figure 3, Figure 4, Figure 5 and Figure S2). The improved restoration outcomes likely reflect restoration strategies tailored to local ecological conditions. For example, restoration at Chongming Dongtan explicitly incorporated habitat requirements of multiple waterbird guilds, including shorebirds, by creating a diverse mosaic of habitat types [39]. Increased habitat heterogeneity is known to support more diverse waterbird communities [39], which may partly explain the marked post-restoration increases in richness and abundance observed for some groups. In addition, the eradication of *Spartina alterniflora* was likely a key driver of habitat recovery for waterbirds [40,41]. Notably, Herons appeared highly adaptable to intensively managed artificial wetlands, as their species richness and abundance showed no significant difference between artificial and natural wetlands (Figure 7), further highlighting the role of artificial wetlands as critical supplementary habitats for certain waterbird groups [42].

At the landscape scale, an appropriate spatial configuration of habitats can further mitigate the negative impacts of wetland degradation. Restored wetlands with high habitat heterogeneity not only increase species richness, abundance, and diversity indices of bird communities, but also provide critical habitats for threatened species. Cheng et al. (2022) reported that more than 50% of threatened waterbird individuals were recorded in restored wetlands [39], underscoring their substantial conservation value. In addition, restoration projects may generate unexpected ecosystem services, such as providing summer breeding habitats for certain waterbird species, further highlighting the multifunctional benefits of restoration [43]. Our study suggests that wetland restoration not only effectively enhances waterbird diversity, but also delivers broad conservation benefits through habitat optimization and landscape integration, particularly by supporting threatened species and expanding ecosystem service provision [44,45].

Our results suggest that the artificial wetland restoration at Chongming Dongtan, Shanghai, represents a highly successful model of coastal wetland restoration. This is particularly notable given the well-documented long-term declines in China’s waterbird populations and the marked reduction in wetland bird diversity observed in the lower Yangtze River Basin [46,47]. Chongming Dongtan is therefore of critical importance for sustaining bird diversity in the middle and lower Yangtze region and along the East Asian–Australasian Flyway, with broader implications for global biodiversity conservation [48,49,50,51,52].

### 4.2. Landscape-Scale Spillover Effects of Wetland Restoration

One well-supported mechanistic explanation is that restored wetlands provide resources (e.g., food and suitable space) that can spread to nearby non-restored mudflats, generating a “spillover effect” [15]. Similar “small-area, large-effect” outcomes have been reported in other ecosystems [53,54], including spillover of fish biomass from marine protected areas and spillover benefits of pollination services in agricultural landscapes [55,56], where introducing small flower-rich patches can substantially increase pollinator abundance both within restored sites and in surrounding non-restored areas [57,58,59,60]. Meta-analyses further suggest that small habitat patches (e.g., localized restoration sites) can contribute disproportionately to regional biodiversity relative to their spatial extent, and may be critical for maintaining landscape-scale diversity [61,62,63]. Nevertheless, spillover effects are not universally positive; their ecological effects can be either beneficial or detrimental depending on context [61]. Even when overall habitat quality is high, local degradation can still exert strong negative impacts on species and communities [5,16,23].

Previous studies have demonstrated that large-scale and high-intensity human activities have caused severe loss and degradation of coastal wetlands, which, in turn, have driven population declines in many waterbird species [7,64]. A systematic assessment of 276 biogeographic populations belonging to 216 species within the East Asian–Australasian Flyway (EAAF) indicated that the overall trend of waterbird populations in this region is dominated by declines: among the 159 populations with well-documented change trends, 67 (42%) are declining, 48 (30%) are stable, and only 44 (28%) are increasing [7,8,9]. Along the EAAF, many critical stopover sites are also experiencing rapid declines in waterbird populations, most notably the Yellow Sea tidal mudflats [1]. Consistent with this flyway-wide pattern, a national assessment of population changes in 260 waterbird species across China found that 84 species exhibit declining population trends [20]. Among these declining species, 28.6% are primarily distributed in coastal wetlands, and an additional 32.5% occur in both coastal and inland wetlands [20]. Although active restoration was implemented only in parts of the Chongming Dongtan Bird Nature Reserve (Figure 1), increases in both species richness and individual abundance were observed not only within the restored areas but also in non-restored natural tidal flats (Figure 2, Figure 3, Figure 4 and Figure 5) and across the entire reserve (Figures S1 and S2). This pattern suggests that the ecological benefits of wetland restoration can extend beyond project boundaries and enhance biodiversity at the landscape scale [53,54,55]. Our results contrast with the regional context of declining continental waterbird populations. Waterbird abundance increased in the unrestored natural mudflats of Chongming Dongtan wetlands, which is incompatible with broad-scale regional recovery as the primary driver (Figure 2 and Figure S1), and supports the spillover interpretation that local wetland restoration, not regional population changes, is responsible for the observed patterns.

Our results suggest that ecological restoration may generate substantial landscape-scale benefits. We found that post-restoration artificial wetlands supported significantly higher individual abundances (Figure 2). Interestingly, species richness in nearby non-restored natural wetlands also increased post-restoration (Figure 3, Figure 4 and Figure 5), although the magnitude of this increase was lower than that observed in restored artificial wetlands (Table 1). This pattern suggests that the positive effects of wetland restoration may extend to, and benefit, surrounding wetland ecosystems that have not directly undergone restoration [65]. Successful restoration projects can enhance biodiversity within restored patches while also benefiting adjacent areas through mechanisms such as providing sources of colonizing individuals, improving local microclimates, and strengthening landscape connectivity [66]. As restored ecosystems progressively recover ecological complexity—such as food-web structure and species interactions [34,36,37,38]—restoration benefits may further expand across both time and space [65,66]. The landscape context of restored habitats is, therefore, crucial: species from surrounding, less-degraded habitats can spill over into newly created or improved habitats, thereby amplifying restoration outcomes [67,68,69]. Embedding the concept of landscape-scale benefits more firmly into restoration practice may help improve project design, optimize spatial configuration, and ultimately increase overall ecological effectiveness [56,57,58].

### 4.3. Seasonal and Guild-Specific Habitat Use After Wetland Restoration

Following restoration, overall waterbird abundance increased significantly, and drove pronounced seasonal shifts in habitat use among waterbird groups. Both the composition of waterbird guilds supported by the wetland and their relative contributions to total abundance varied across seasons and restoration phases. During the spring northward and autumn southward migrations, the reserve was dominated by shorebirds (Waders), which accounted for 56.4–82.5% of individuals in spring (Figure 6a) and 46.7–64.5% of individuals in autumn (Figure 6b). In contrast, during the wintering period, Anatidae became the predominant group, comprising 46.1–74.3% of individuals (Figure 6c).

Water-level management in artificial wetlands creates foraging opportunities for birds of different ecological types [22,36,70]. Within artificial wetlands, aquaculture ponds and their seasonal production activities can provide additional foraging opportunities. For example, in autumn, lowering pond water levels can expose mudflats that attract Waders, Gulls, and Herons to feed on small fish, shrimp, and invertebrates left after harvesting [71]. In addition, along coastal zones, artificial wetlands can serve as critical high-tide refuges or stopover habitats for shorebirds during high tides [49,50,51]. Together, these findings further support the view that restored and managed artificial wetlands can function as critical supplementary habitats for waterbirds [7,33,34,51], thereby making a substantial contribution to the long-term maintenance of regional waterbird diversity [23,56]. More broadly, agricultural landscapes (including aquaculture ponds) often provide highly productive but short-lived food resources due to frequent human management. Such transient resources can attract Anatidae, shorebirds, Gulls, and Herons/egrets to concentrate in artificial wetlands during particular seasons, leading to pronounced seasonal fluctuations in habitat use [72].

Successful long-distance migration depends critically on the spatiotemporal continuity of suitable habitats along migratory routes. Accordingly, restoration projects that maintain or create a heterogeneous mosaic of habitat types—and thereby restore ecosystem structure and function to meet the requirements of multiple waterbird guilds—are likely to deliver sustained ecological benefits and long-term conservation benefits. A well-designed landscape configuration is critical for sustaining regional biodiversity and ecosystem functioning [67].

### 4.4. Short-Term Disturbance but Long-Term Benefits

Restoration activities can cause temporary, localized disturbance to waterbird communities; however, our results suggest that such effects are short-lived and fully reversible, and that the long-term benefits substantially exceed any short-term negative impacts [70,71,72]. The detection rate of the threatened species temporarily declined during the construction phase (from 82.6% in pre-restoration to 52.2%), showing that human activities can displace sensitive waterbird species. However, there is a rapid rebound in detection rates (91.3%) in the post-restoration phase. Construction and associated human activities during the restoration period likely caused transient disturbance to some rare species (e.g., Little Curlew and Siberian Crane), which were not recorded in the reserve during this phase (Figure 8). Importantly, these adverse effects disappeared after construction ended: in the post-restoration phase, previously recorded species reappeared, and additional threatened species—such as the Spoon-Billed Sandpiper—were detected, potentially indicating successful recovery of habitat functions (Figure 8). Moreover, at the community level, waterbird abundance was not markedly suppressed during the restoration period; most groups remained stable or increased (Figure 2, Figure 3, Figure 4 and Figure 5). From the perspective of the core conservation objective—supporting threatened species—this project represents a highly successful model of coastal wetland restoration. Nevertheless, it should be emphasized that although artificial wetlands can effectively supplement habitats for waterbirds, their ecological functions cannot fully compensate for the irreversible loss of natural wetland ecosystems.

### 4.5. Study Limitations

This study focused primarily on changes in waterbird species richness and abundance, and did not incorporate functional trait analyses or explicitly evaluate species’ ecological roles and the ecological roles of individual species and their responses to fine-scale environmental change. Changes in artificial wetland area may interfere with population patterns, and *Spartina alterniflora* restoration represents only one primary influencing factor. Consequently, our assessment of restoration outcomes is largely restricted to community-level diversity patterns. To provide stronger, more actionable support for evidence-based wetland restoration practice, more comprehensive and representative evaluation frameworks are needed. We suggest that future studies integrate multiple trophic levels (e.g., vegetation, fish, benthic fauna, and plankton) through predator–prey linkages into a unified assessment, thereby more fully capturing ecosystem-wide restoration effects. In addition, we did not explicitly disentangle the potential confounding effects of broader-scale drivers such as climate warming or shifts in regional migratory routes; future work could combine remote-sensing indicators (e.g., NDVI) and temperature data to separate these effects. Another limitation is that our pre-restoration baseline (2007–2013) reflects a *Spartina alterniflora*-degraded state (the density of all waterbirds was 6.79 ± 0.59, Figure 2d). Pre-invasion wetlands, dominated by *Scirpus mariqueter*, supported higher waterbird densities (20.00 ± 2.48) [27], and fortunately, the post-restoration density (21.50 ± 2.40, Figure 2d) nearly returned to this historical condition. Nevertheless, the positive trends documented here suggest that ecological restoration at Chongming Dongtan has begun to deliver tangible ecological benefits and that its overall ecological value may extend well beyond what is captured in the present study.

## 5. Conclusions

Our study suggests that large-scale control of *Spartina alterniflora* and habitat improvement at Chongming Dongtan have generated substantial, long-term conservation benefits for waterbird conservation. Wetland restoration increased overall waterbird abundance and species richness, supported threatened species, and enhanced habitat use by different waterbird guilds across seasons. Importantly, restoration effects extended beyond restored patches, with increased waterbird diversity also detected in adjacent, non-restored natural mudflats, indicating a landscape-scale spillover effect. These findings highlight the value of incorporating habitat heterogeneity, seasonal habitat requirements, and landscape context into coastal wetland restoration and management strategies.

## Figures and Tables

**Table 1:** Effect sizes (*η*^2^) from Kruskal–Wallis tests for differences in bird abundance.

	Spring		Autumn		Winter	
	Artificial Wetlands	Natural Wetlands	Artificial Wetlands	Natural Wetlands	Artificial Wetlands	Natural Wetlands
Anatidae	0.59 **	0.56 **	0.72 **	−0.14	0.73 **	0.01
Waders	0.33 *	−0.14	0.51 *	0.72 **	0.11	−0.02
Gulls	0.56 **	0.14	0.75 **	−0.02	−0.07	0.19
Herons	0.69 **	0.41 *	0.74 **	−0.04	−0.11	−0.11

Note: Negative effect sizes (*η*^2^) were derived from bias-corrected epsilon-squared estimates, indicating extremely small and negligible effect sizes. * significant difference; ** highly significant difference.

## Data Availability

The data presented in this study are available on request from the corresponding author due to privacy restrictions.

## Supplementary Materials

The following supporting information can be downloaded at https://www.mdpi.com/article/10.3390/biology15120926/s1. Figure S1: The species richness and population from 2007 to 2023 in Chongming Dongtan National Nature Reserve; Figure S2: Variation in species richness of different groups across restoration periods.
