# Supplementary material for: Wetland Restoration Effects on Waterbird Diversity and Habitat Use: A Long-Term Case Study from Chongming Dongtan in Shanghai, China
Source: Biology (Basel). 2026 Jun 13;15(12):926. doi: 10.3390/biology15120926 (PMC13296075; doi:10.3390/biology15120926)
Supplement: Supplementary file 1 [file biology-15-00926-s001.zip › biology-4317341-supplementary.pdf]

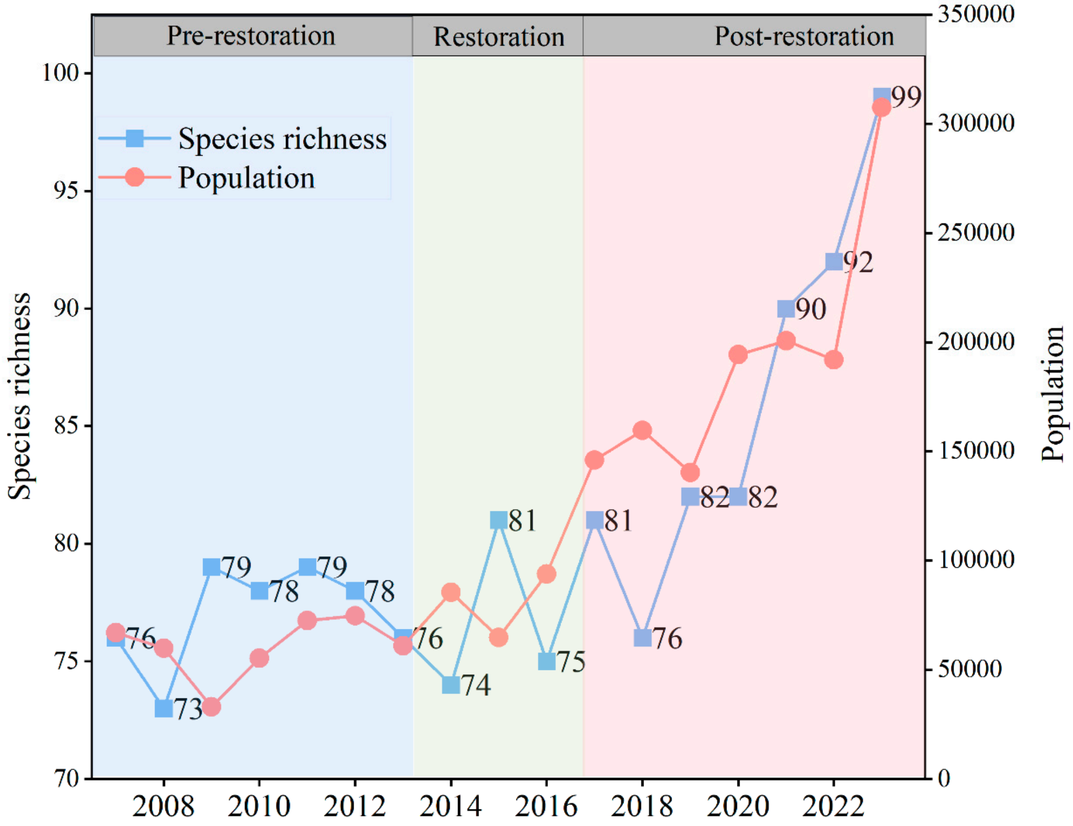

Fig S1 The species richness and population from 2007 to 2023 in Chongming Dongtan National Nature Reserve

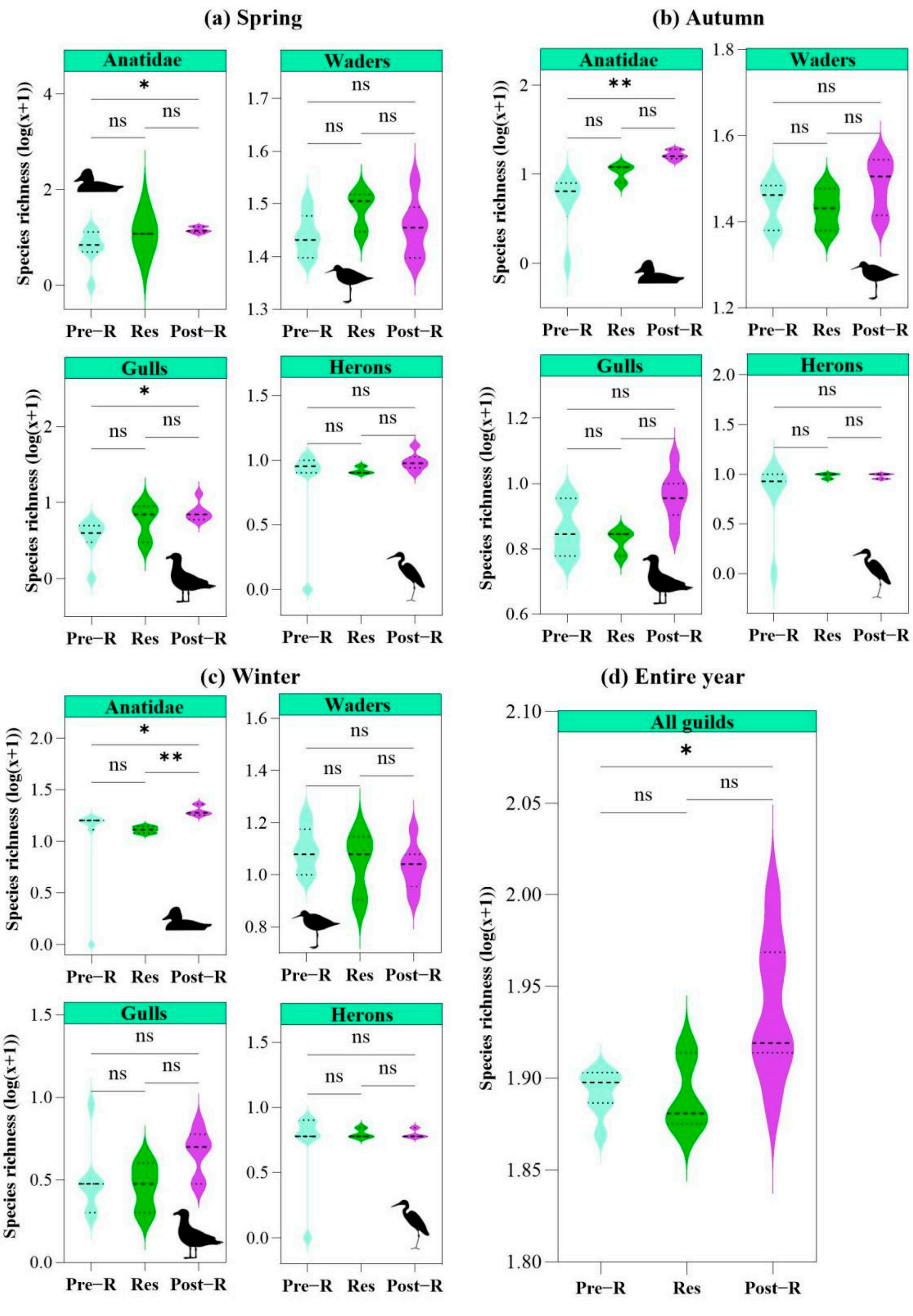

Fig S2 Variation in species richness of different groups across restoration periods (Pre−R: 2007-2013; Res: 2014-2016; Post−R: 2017-2023. * $p < 0.05$， ** $p < 0.01$， ns $p > 0.05$)
